# Enhancing surface heat transfer by carbon nanofins: towards an alternative to nanofluids?

**DOI:** 10.1186/1556-276X-6-249

**Published:** 2011-03-22

**Authors:** Eliodoro Chiavazzo, Pietro Asinari

**Affiliations:** 1 Department of Energetics, Politecnico di Torino, Corso Duca degli Abruzzi, 10129 Torino, Italy

## Abstract

**Background:**

Nanofluids are suspensions of nanoparticles and fibers which have recently attracted much attention because of their superior thermal properties. Nevertheless, it was proven that, due to modest dispersion of nanoparticles, such high expectations often remain unmet. In this article, by introducing the notion of *nanofin*, a possible solution is envisioned, where nanostructures with high aspect-ratio are sparsely attached to a solid surface (to avoid a significant disturbance on the fluid dynamic structures), and act as efficient thermal bridges within the boundary layer. As a result, particles are only needed in a small region of the fluid, while dispersion can be controlled in advance through design and manufacturing processes.

**Results:**

Toward the end of implementing the above idea, we focus on single carbon nanotubes to enhance heat transfer between a surface and a fluid in contact with it. First, we investigate the thermal conductivity of the latter nanostructures by means of classical non-equilibrium molecular dynamics simulations. Next, thermal conductance at the interface between a single wall carbon nanotube (nanofin) and water molecules is assessed by means of both steady-state and transient numerical experiments.

**Conclusions:**

Numerical evidences suggest a pretty favorable thermal boundary conductance (order of 10^7 ^W·m^-2^·K^-1^) which makes carbon nanotubes potential candidates for constructing *nanofinned surfaces*.

## Background and motivations

Nanofluids are suspensions of solid particles and/or fibers, which have recently become a subject of growing scientific interest because of reports of greatly enhanced thermal properties [1,2]. Filler dispersed in a nanofluid is typically of nanometer size, and it has been shown that such nanoparticles are able to endow a base fluid with a much higher effective thermal conductivity than fluid alone [3,4]: significantly higher than those of commercial coolants such as water and ethylene glycol. In addition, nanofluids show an enhanced thermal conductivity compared to theoretical predictions based on the Maxwell equation for a well-dispersed particulate composite model. These features are highly favorable for applications, and nanofluids may be a strong candidate for new generation of coolants [2]. A review about experimental and theoretical results on the mechanism of heat transfer in nanofluids can be found in Ref. [5], where those authors discuss issues related to the technology of nanofluid production, experimental equipment, and features of measurement methods. A large degree of randomness and scatter has been observed in the experimental data published in the open literature. Given the *inconsistency *in these data, we are unable to develop a comprehensive physical-based model that can predict all the experimental evidences. This also points out the need for a systematic approach in both experimental and theoretical studies [6].

In particular, carbon nanotubes (CNTs) have attracted great interest for nanofluid applications, because of the claims about their exceptionally high thermal conductivity [7]. However, recent experimental findings on CNTs report an *anomalously *wide range of enhancement values that continue to perplex the research community and remain unexplained [8]. For example, some experimental studies showed that there is a modest improvement in thermal conductivity of water at a high loading of multi-walled carbon nanotubes (MW-CNTs), approximately of 35% increase for a 1 wt% MWNT nanofluid [9]. Those authors attribute the increase to the formation of a nanotube network with a higher thermal conductivity. On the contrary, at low nanotube content, <0.03 wt%, they observed a decrease in thermal conductivity with an increase of nanotube concentration. On the other hand, more recent experimental investigations showed that the enhancement of thermal conductivity as compared with water varied linearly when MW-CNT weight content was increased from 0.01 to 3 wt%. For a MWNT weight content of 3 wt%, the enhancement of thermal conductivity reaches 64% of that of the base fluid (e.g., water). The average length of the nanotubes appears to be a very sensitive parameter. The enhancement of thermal conductivity compared with water alone is enhanced when nanotube average length is increased in the 0.5-5 *μ*m range [10].

**Figure 1:**
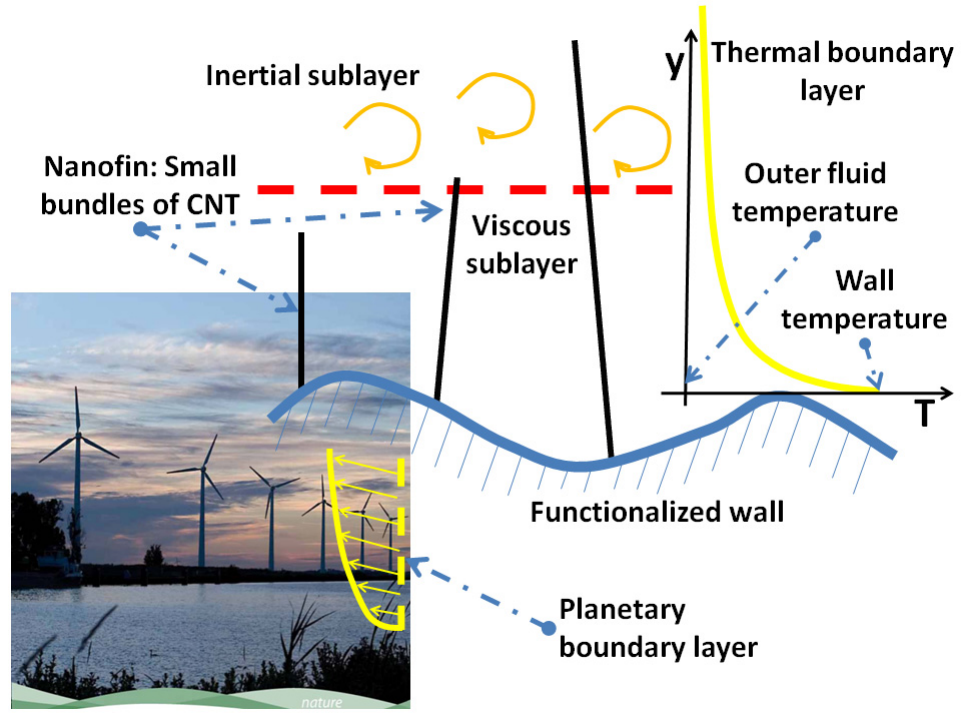
**Color online. Eolic parks represent a macroscopic analogy of the proposed *nanofin *concept: wind towers are slim enough to avoid disturbing the planetary boundary layer, but high enough to reach the region where wind is stronger**. Similarly nanofins do not interfere with the thermal boundary layer, but they allow direct energy transfer between the wall and the bulk fluid, thus acting as thermal bridges. The picture of the wind farm is provided as courtesy of the European Commission, October 2010: *EU Guidance on wind energy development in accordance with the EU nature legislation*.

Clearly, there are difficulties in the experimental measurements [11], but the previous results also reveal some underlaying technological problems. First of all, the CNTs show some bundling or the formation of aggregates originating from the fabrication step. Moreover, it seems reasonable that CNTs encounter *poor dispersibility *and suspension durability because of the aggregation and surface hydrophobicity of CNTs as a nanofluid filler. Therefore, the surface modification of CNTs or additional chemicals (surfactants) have been required for stable suspensions of CNTs, because of the polar characteristics of base fluid. In the case of surface modification of CNTs, water-dispersible CNTs have been extensively investigated for potential applications, such as biological uses, nanodevices, novel precursors for chemical reagents, and nanofluids [2]. From the above brief review, it is possible to conclude that, despite the great interest and intense research in this field, the results achieved so far cannot be considered really encouraging. Hence, toward the end of overcoming these problems, we introduce the notion of *thermal nanofins*, with an entirely different meaning with respect to standard terminology. By nanofins, we mean slender nano-structures, sparse enough not to interfere with the thermal boundary layer, but sufficiently rigid and conductive to allow for direct energy transfer between the wall and the bulk fluid, thus acting as thermal bridges. A macroscopic analogy is given by an eolic park, where wind towers are slim enough to avoid disturbing the planetary boundary layer, but high enough to reach the region where the wind is stronger (see Figure 1). In this way, nanoparticles are used only where they are needed, namely, in the thermal boundary layer (or in the thermal laminar sub-layer, in case of turbulent flows, not discussed here), and this might finally unlock the enormous potential of the basic idea behind nanofluids.

This article investigates a possible implementation of the above idea using CNTs, because of their unique geometric features (slimness) and thermo-physical properties (high thermal conductivity). CNTs have attracted the attention of scientific community, since their mechanical and transport (both electrical and thermal) properties were proven to be superior compared with traditional materials. This observation has motivated intensive theoretical and experimental efforts during the last decade, toward the full understanding/exploitation of these properties [12-16]. Despite these expectations, however, it is reasonable to say that these efforts are far from setting out a comprehensive theoretical framework that can clearly describe these phenomena. First of all, the vast majority of CNTs (mainly multi-walled) exhibits a metallic behavior, but the phonon mechanism (lattice vibrations) of heat transfer is considered the prevalent one close to room temperature [17,18]. The phonon mean free path, however, is strongly affected by the existence of lattice defects, which is actually a very common phenomenon in nanotubes and closely linked to manufacturing methods. Second, there is the important issue of quantifying the interface thermal resistance between a nanostructure and the surrounding fluid, which affects the heat transfer and the maximum efficiency. It is noted that, according to the classical theory, there is an extremely low thermal resistance when one reduces the characteristic size of the thermal "antenna" promoting heat transfer [19], as confirmed by numerical investigations for CNTs [20-22].

This article investigates, by molecular mechanics based on force fields (MMFF), the thermal performance of nanofins made of single wall CNTs (SW-CNTs). The SW-CNTs were selected mainly because of time constraints of our parallel computational facilities. The following analysis can be split into two parts. First of all, the heat conductivity of SW-CNTs is estimated numerically by both simplified model (section "Heat conductivity of single-wall carbon nanotubes: a simplified model", where this approach is proved to be inadequate) and a detailed three-dimensional model (section "Heat conductivity of single-wall carbon nanotubes: detailed three dimensional models"). This allows one to appreciate the role of model dimensionality (and harmonicity/anharmonicity of interaction potentials) in recovering standard heat conduction (i.e., Fourier's law). This first step is used for validation purposes in a vacuum and for comparison with results from literature. Next, the thermal boundary conductance between SW-CNT and water (for the sake of simplicity) is computed by two methods: the steady-state method (section "Steady-state simulations"), mimicking ideal cooling by a strong forced convection (thermostatted surrounding fluid), and the transient method (section "Transient simulations"), taking into account only atomistic interactions with the local fluid (defined by the simulation box). This strategy allows one to estimate a reasonable range for the thermal boundary conductance.

## Heat conductivity of SW-CNTs: a simplified model

**Figure 2:**
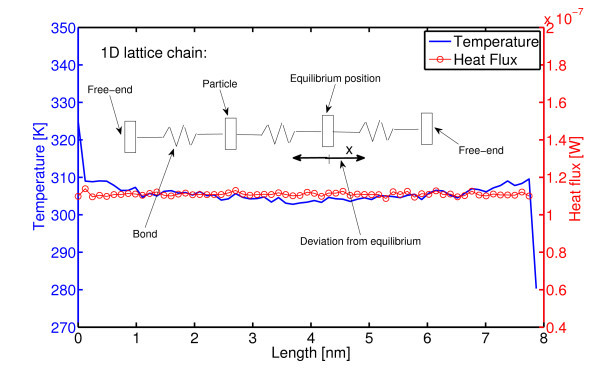
**Color online. One-dimensional model: lattice chain of particles in interaction according to a Morse-type potential (1)**. End-particles are coupled to Nosé-Hoover thermostats at different temperatures (*T*_hot _= 320 K and *T*_cold _= 280 K). Despite of the anharmonicity of the potential, normal heat conduction (Fourier's law) could not be established. In this case, heat flux is computed by Equation (7). However, consistent results are obtained based on Equation (12) which predicts 〈*ξ*_hot_〉 *k*_b_*T*_hot _= - 〈*ξ*_cold_〉 *k*_b_*T*_cold _= 1.11 × 10^-7 ^W.

**Figure 3:**
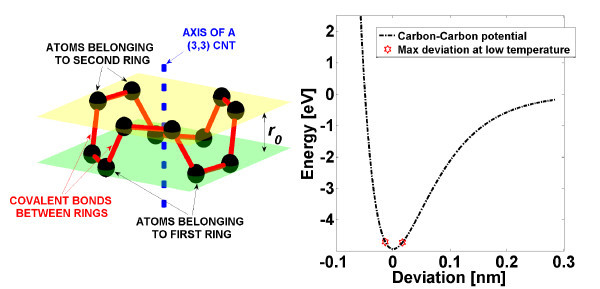
**Color online. Left-hand side: according to the one dimensional model described in section, a single particle is formed by several carbon atoms lying on the same plane orthogonal to the CNT axis**. Particles are linked by means of several carbon-carbon covalent bonds (not aligned with the CNT axis), with *r*_0 _denoting the spacing between particles at rest. Right-hand side: at low temperature, *T <*1000 K, small deviations from the rest position are observed so that the adopted potential (1) can be safely approximated by harmonic Taylor expansion about *x *= 0.

In order to significantly downgrade the difficulty of studying energy transport processes within a CNT, some authors often resort to simplified low-dimensional systems such as one-dimensional lattices [23-28]. In particular, heat transfer in a lattice is typically modeled by the vibrations of lattice particles interacting with the nearest neighbors and by a coupling with thermostats at different temperatures. The latter are the popular *numerical experiments *based on non-equilibrium molecular dynamics (NEMD). In this respect, to the end of measuring the thermal conductivity of a single wall nanotube (SWNT), we set up a model for solving the equations of motion of the particle chain pictorially reported in Figure 2 where each particle represents a ring of several atoms in the real nanotube (see also the left-hand side of Figure 3). In the present model, carbon-carbon-bonded interactions between *first neighbors *(i.e., atoms of the *i*th particles and atoms of the particles *i *± 1) separated by a distance *r *are taken into account by a Morse-type potential (shown on the right-hand side of Figure 3) [29] expressed in terms of deviations *x *= *r *- *r*_0 _from the bond length *r*_0_:(1)

where *V*_0 _is the bond energy, while *a *is assumed as *a *= *r*_0_/2. Following [30], the bond energy *V*_0 _= 4.93 eV, while the distance between two consecutive particles at equilibrium is assumed as *r*_0 _= 0.123 nm. At any arbitrary configuration, the total force, *F_i _*, acting on the *i*th particle is computed as(2)

with d*x*_*i*-*j *_= *x_i _*- *x*_*i*-*j*_, d*x*_*i*+*j *_= *x*_*i*+*j *_- *x_i_*, and *N*_bon _denoting the number of carbon-carbon bonds between two particles, whereas a penalization factor sin ϑ may be included to account for bonds not aligned with the tube axis (see Figure 3). In the present case, we use free-end boundary condition, and hence, forces experienced by particles at the ends of the chain read:(3)

Let *p_i _*and *m_i _*be the momentum and mass of the *i*th particle, respectively; the equations of motion for the inner particles take the form:(4)

whereas the outermost particles (*i *= 1, *N *) are coupled to Nosé-Hoover thermostats and are governed by the equations:(5)

with *k*_b_, *T*_0_, *N*_f_, and *τ*_T _denoting the Boltzmann constant, the thermostat temperature, number of degrees of freedom, and relaxation time, respectively, while the auxiliary variable *ξ *is typically referred to as *friction coefficient *[31]. Nosé-Hoover thermostatting is preferred since it is deterministic and it typically preserves canonical ensemble. However, we notice that (5) represent the equations of motions with a single thermostat. In this case, it is known that the latter scheme may run into ergodicity problems and thus fail to generate a canonical distribution. Although stochastic thermostats (see, e.g., Andersen [32]) are purposely devised to generate a canonical distribution, they are characterized by a less realistic dynamics. Hence, to the end of overcoming the above issues, using deterministic approaches, Martyna et al. have introduced the idea of Nosé-Hoover chain [33] (see also [34,35] for the equation of motion of Nosé-Hoover chains and further details on thermostats in molecular dynamics simulations). Simulations presented below were carried out using both a single thermostat and a Nosé-Hoover chain (with two thermostats), and no differences were noticed.

Local temperature *T_i_*(*t*) at a time instant *t *is computed for each particle *i *using energy equipartition:(6)

where 〈〉 denotes time averaging. On the other hand, local heat flux *J_i_*, transferred between particle *i *and *i *+ 1, can be linked to mechanical quantities by the following relationship [25,27]:(7)

The above simplified model has been tested in a range of *low *temperature (300 K *< T <*1000 K), where we notice that it is not suitable to predict normal heat conduction (Fourier's law). In other words, at steady state (i.e., when heat flux is uniform along the chain and constant in time) is observed a finite heat flux although no meaningful temperature gradient could be established along the chain (see Figure 2). Thus, the above results predict a divergent heat conductivity. In this context, it is worth stressing that one-dimensional lattices with harmonic potentials are known to violate Fourier's law, and they exhibit a flat temperature profile (and divergent heat conductivity). On the one hand, the results of the simplified model in Figure 2 are likely due to a non-sufficiently strong anharmonicity. Indeed, as reported on the right-hand side of Figure 3, the Morse function (1) can be safely approximated by an harmonic potential in the range of maximal deviation *x *observed at low temperature (*T <*1000 K), namely, *V*_b _(*x*) ≈ *V*_0 _(*x*^2^/*a*^2 ^- 1).

On the other hand, it is worth stressing that it has been demonstrated that anharmonicity alone is insufficient to ensure normal heat conduction [23], in one-dimensional lattice chains.

## Heat conductivity of SW-CNTs: detailed three-dimensional models

In all simulations below, we have adopted the open-source molecular dynamics (MD) simulation package GROningen MAchine for Chemical Simulations (GROMACS) [36-38] to investigate the energy transport phenomena in three-dimensional SWNT obtained by a freely available structure generator (Tubegen) [39]. Three harmonic terms are used to describe the carbon-carbon-bonded interactions within the SWNT. That is, a bond stretching potential (between two covalently bonded carbon atoms *i *and *j *at a distance *r_ij_*):(8)

a bending angle potential (between the two pairs of covalently bonded carbon atoms (*i*, *j*) and (*j*, *k*))(9)

and the Ryckaert-Bellemans potential for proper dihedral angles (for carbon atoms *i*, *j*, *k *and *l*)(10)

are considered in the following MD simulations. In this case, *θ_ijk _*and *ϕ_ijkl _*represent all the possible bending and torsion angles, respectively, while  = 0.142 and  = 120° are the reference geometry parameters for graphene. Non-bonded van der Waals interaction between two individual atoms *i *and *j *at a distance *r_ij _*can be also included in the model by a Lennard-Jones potential:(11)

where the force constants ,  and  in (8), (9), and (10) and the parameters (*σ*_CC_, ϵ_CC_) in (11) are chosen according to the Table 1 (see also [40,41]). In reversible processes, differentials of heat d*Q*_rev _are linked to differentials of a state function, entropy, d*s *through temperature: d*Q*_rev _= *T *d*s*. Moreover, following Hoover [31,42], entropy production of a Nosé-Hoover thermostat is proportional to the time average of the friction coefficient 〈*ξ*〉 through the Boltzmann constant *k*_b_, and hence, once a steady-state temperature profile is established along the nanotube, the heat flux per unit area within the SWNT can be computed as(12)

**Table 1:** Parameters for carbon-carbon, carbon-water, and water-water interactions are chosen according to Guo et al. [40] and Walther et al. [41]

Carbon-carbon interactions	
	47890 kJ·mol^-1^·nm^-2^
	562.2 kJ·mol^-1^
	25.12 kJ·mol^-1^
ϵ_CC_	0.4396 kJ·mol^-1^
*σ*_CC_	3.851 Å

**Carbon-oxygen interactions**	

ϵ_CO_	0.3126 kJ·mol^-1^
*σ*_CO_	3.19 Å

**Oxygen-oxygen interactions**	

ϵ_OO_	0.6502 kJ·mol^-1^
*σ*_OO_	3.166 Å

**Oxygen-hydrogen interactions**	

*q*_O_	-0.82e
*q*_H_	0.41 e

where the cross section *S*_A _is defined as *S*_A _= 2*πrb*, with *b *= 0.34 nm denoting the van der Waals thickness (see also [43]). In this case, the use of formula (12) is particularly convenient since the quantity 〈*ξ*〉 can be readily extracted from the output files in GROMACS.

**Figure 4:**
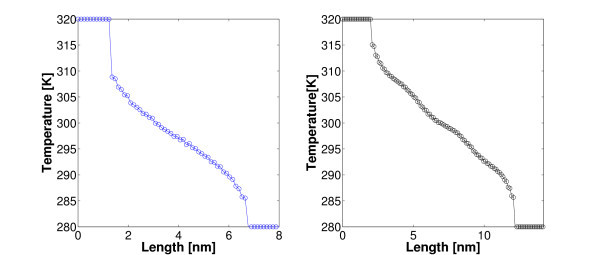
**Color online. Three-dimensional model: Nosé-Hoover thermostats are coupled to the end atoms of a (5, 5) SWNT**. Both bonded (8), (9), and (10), and non-bonded interactions (11) are considered. In a three-dimensional structure, harmonic-bonded potentials do give rise to normal heat conduction. Temperature profiles for two lengths (5.5 and 10 nm) are reported.

**Figure 5:**
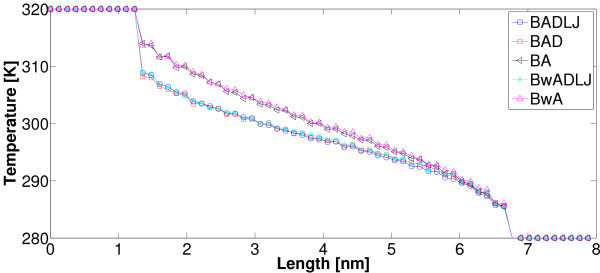
**Color online. Several setups have been tested where some of the interaction potentials (8), (9), (10), and (11) are omitted**. BADLJ: *V*_b_, *V*_an_, *V*_rb_, and *V*_nb _are considered. BAD: *V*_b_, *V*_an_, *V*_rb _are considered. BA: *V*_b _and *V*_an _are considered. Bw denotes that *V*_b _is computed with a smaller force constant  = 42000 kJ·mol^-1^·nm^-2 ^according to [30].

The measure of both the slopes of temperature profiles along the inner rings of SWNT in Figures 4 and 5 and the heat flux by (12) enables us to evaluate heat conductivity *λ *according to Fourier's law. It is worth stressing that, as shown in the latter figures, unlike one-dimensional chains such as the one discussed above, fully three-dimensional models do predict normal heat conduction even when using harmonic potentials such as (8), (9), and (10). Nevertheless, we notice that in the above three-dimensional model, anharmonicity (necessary condition for standard heat conduction in one-dimensional lattice chains [23]), despite the potential form itself, intervenes due to a more complicated geometry and the presence of angular and dihedral potentials (9), and (10). Interestingly, in our simulations we can omit at will some of the interaction terms *V*_b_, *V*_a_, *V*_rb_, and *V*_nb_, and investigate how temperature profile and thermal conductivity *λ *are affected. It was found that potentials *V*_b _and *V*_a _are strictly needed to avoid a collapse of the nanotube. Results corresponding to several setups are reported in Figure 5 and Table 2. It is worth stressing that, for all simulations in a vacuum, non-bonded interactions *V*_nb _proven to have a negligible effect on both the slope of temperature profile and heat flux at steady state. On the contrary, the torsion potential *V*_rb _does have impact on the temperature profile while no significant effect on the heat flux was noticed: as a consequence, in the latter case, thermal conductivity shows a significant dependence on *V*_rb_. More specifically, the higher the torsion rigidity the flatter the temperature profile. Depending on the CNT length (and total number of atoms), computations were carried out for 4 ns up to 6 ns to reach a steady state of the above NEMD simulations. Finally, temperature values of the end-points of CNTs (see Figures 4, 5) were chosen following others [16,18].

**Table 2:** Summary of the results of MD simulations in this study.

Chirality, case	Box	***L***_**NH**_	*L*	*λ*	***α***_**st**_	***α***_**tr**_	***τ***_**d**_	*mL/2*
	**(nm**^**3**^)	(nm)	(nm)	**W·m**^**-2**^**·K**^**-1**^	**W·m**^**-2**^**·K**^**-1**^	**W·m**^**-2**^**·K**^**-1**^	(ps)	
(5, 5), BAD-LJ (vac)	12 × 12 × 12	1.5	5.5	67	-	-	-	-
(5, 5), BwAD-LJ (vac)	12 × 12 × 12	1.5	5.5	64	-	-	-	-
(5, 5), BAD (vac)	12 × 12 × 12	1.5	5.5	65	-	-	-	-
(5, 5), BA (vac)	12 × 12 × 12	1.5	5.5	49	-	-	-	-
(5, 5), BwA (vac)	12 × 12 × 12	1.5	5.5	48.9	-	-	-	-
(5, 5), BAD-LJ (vac)	20 × 20 × 20	2	10	96.9	-	-	-	-
(5, 5), BAD-LJ (vac)	105 × 105 × 105	25	25	216.1	-	-	-	-
(5, 5), BAD-LJ (sol)	2.5 × 2.5 × 14	2	10	-	5.18 × 10^7^	-	-	0.28
(5, 5), BAD-LJ (sol)	4 × 4 × 14	2	10	-	5.18 × 10^7^	-	-	0.28
(5, 5), BAD-LJ (sol)	4 × 4 × 14	0	14	-	-	1.70 × 10^7^	33	-
(5, 5), BAD-LJ (sol)	5 × 5 × 5	0	3.7	-	-	1.37 × 10^7^	41	-
(15, 0), BAD-LJ (sol)	5 × 5 × 5	0	4.7	-	-	1.60 × 10^7^	35	-
(15, 0), BAD-LJ (sol)	5 × 5 × 5	0	3.8	-	-	1.43 × 10^7^	39	-
(3, 3), BAD-LJ (sol)	5 × 5 × 5	0	3.7	-	-	8.90 × 10^6^	63	-

SW-CNTs with chirality (3, 3), (5, 5), and (15, 0) are considered, and several combinations of interaction potentials are tested. In the first column, B, A, D, and LJ stand for bond stretching, angular, dihedrals, and Lennard-Jones potentials, respectively, while Bw denotes bond stretching with a smaller force constant  = 42000 (kJ·mol^-1^·K^-1^) according to [30]. Simulations are carried out both in a vacuum (vac) and within water (sol).

## Thermal boundary conductance of a carbon nanofin in water

### Steady-state simulations

**Figure 6:**
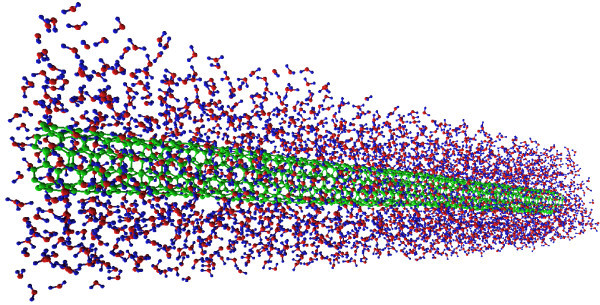
**Color online. A (5, 5) SWNT (green) is surrounded by water molecules (blue, red). Nosé-Hoover thermostats with temperature *T*_hot _= 360 K are coupled to the nanotube tips, while water is kept at a fixed temperature *T*_w _= 300 K**. After a sufficiently long time (here 15 ns), a steady-state condition is reached. MD simulation results (in terms of both temperature profile and heat flux) are consistent with a continuous one-dimensional model as described by Equations (17) and (18). Image obtained using VEGA ZZ [47].

In this section, we investigate on the heat transfer between a carbon nanotube and a surrounding fluid (water). The latter represents a first step toward a detailed study of a batch of single CNTs (or small bundles) utilized as *carbon nanofins *to enhance the heat transfer of a surface when transversally attached to it. To this end, and limited by the power of our current computational facilities, we consider a (5, 5) SWNT (with a length *L *≤ 14 nm) placed in a box filled with water (typical setup is shown in Figure 6). SWNT end temperatures are set at a fixed temperature *T*_hot _= 360 K, while the solvent is kept at *T*_w _= 300 K. The carbon-water interaction is taken into account by means of a Lennard-Jones potential between the carbon and oxygen atoms with a parameterization (ϵ_CO_, *σ*_CO_) reported in Table 1. Moreover, non-bonded interactions between the water molecules consist of both a Lennard-Jones term between oxygen atoms (with ϵ_OO_, *σ*_OO _from Table 1) and a Coulomb potential:(13)

where *ε*_0 _is the permittivity in a vacuum, while *q_i _*and *q_j _*are the partial charges with *q*_O _= -0.82 e and *q*_H _= 0.41 e (see also [41]).

**Figure 7:**
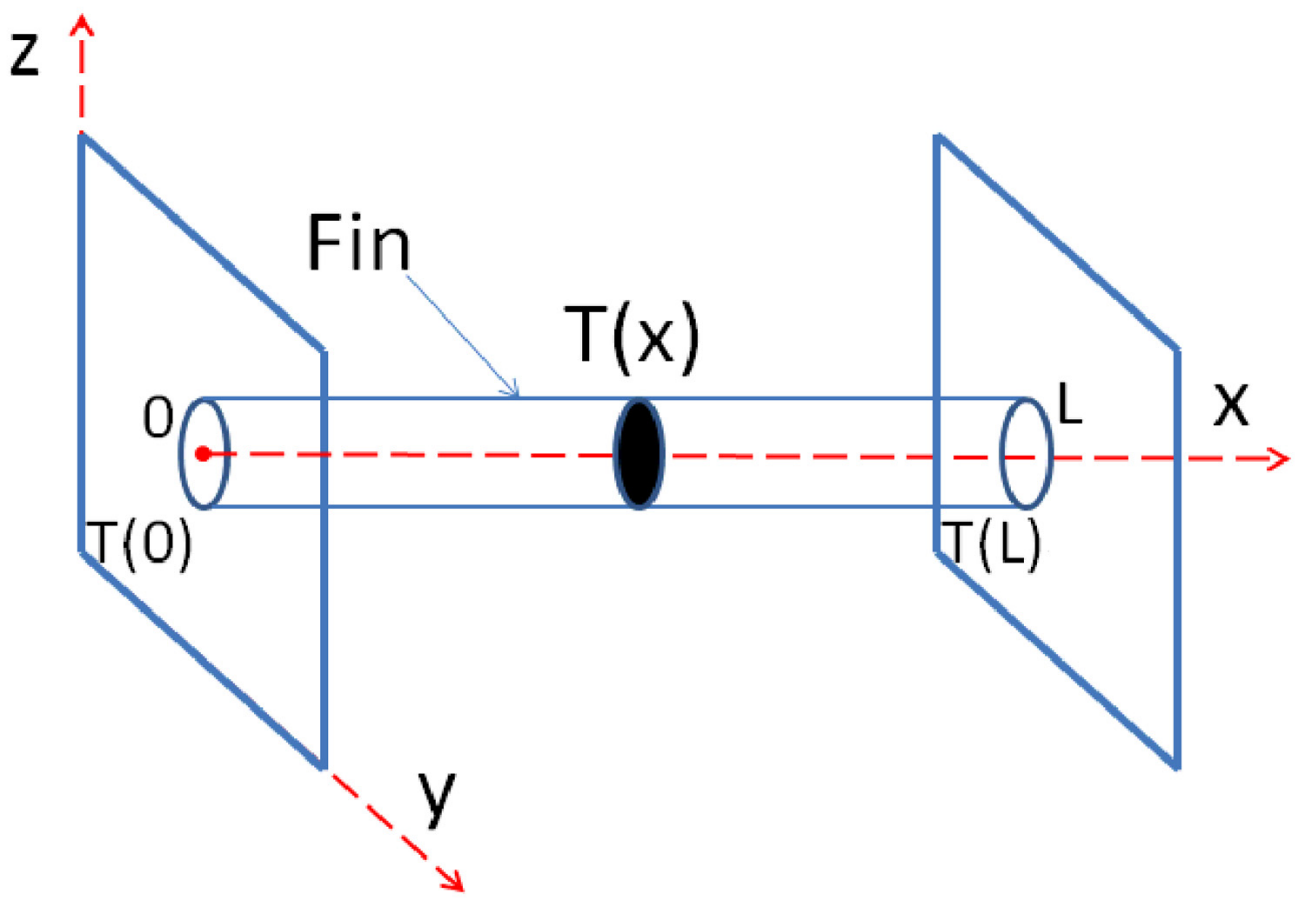
**Color online. Pictorial representation of a single nanofin: end-points are maintained at fixed temperature by Nosé-Hoover thermostats**. During numerical experiments for evaluating thermal conductivity, simulations are conducted in a vacuum. On the contrary, thermal boundary conductances are evaluated with the nanofin surrounded by a fluid. The latter setup can be studied by a one-dimensional continuous model, where all fields are assumed to vary only along the *x*-axis.

We notice that, the latter is a classical problem of heat transfer (pictorially shown in Figure 7), where a single fin (heated at the ends) is immersed in a fluid maintained at a fixed temperature. This system can be conveniently treated using a continuous approach under the assumptions of homogeneous material, constant cross section *S*, and one-dimensionality (no temperature gradients within a given cross section) [44]. In this case, both temperature field and heat flux only depend on the spatial coordinate *x*, and the analytic solution of the energy conservation equation yields, at the steady state, the following relationship:(14)

where  denotes the difference between the local temperature at an arbitrary position *x *and the fixed temperature *T*_w _of a surrounding fluid. Let *α *and *C *be the thermal boundary conductance and the perimeter of the fin cross sections, respectively, *m *be linked to geometry, and material properties as follows:(15)

whereas the two parameters *M *and *N *are dictated by the boundary conditions, *T *(0) = *T *(*L*) = *T*_hot _(or equivalently, due to symmetry, zero flux condition: d*T/*d*x *(*L/*2) = 0), namely:(16)

Thus, the analytic solution (14) takes a more explicit form:(17)

whereas the heat flux at one end of the fin reads:(18)

**Figure 8:**
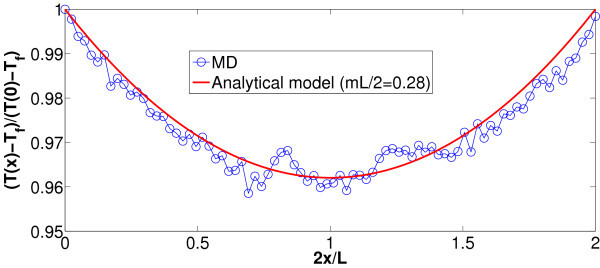
**Color online. Steady-state MD simulations**. Dimensionless temperature computed by MD (symbols) versus temperature profile predicted by continuous model (line), Equation (17). Best fitting is achieved by choosing *mL/*2 = 0.28. Case with computational box 2.5 × 2.5 × 14 nm^3^.

In the setup illustrated in Figures 7 and 6, periodic boundary conditions are applied in the *x*, *y*, and *z *directions, and all the simulations are carried out with a fixed time step d*t *= 1 fs upon energy minimization. First of all, the whole system is led to thermal equilibrium at *T *= 300 by Nosé-Hoover thermostatting implemented for 0.8 ns with a relaxation time *τ*_T _= 0.1 ns. Next, the simulation is continued for 15 ns where Nosé-Hoover temperature coupling is applied only at the tips of the nanofin (here, the outermost 16 carbon atom rings at each end) with *T*_hot _= 360 K, and in water with *T*_w _= 300 K until, at the steady state, the temperature profile in Figure 8 is developed. Moreover, pressure is set to 1 bar by Parrinello-Rahman barostat during both thermal equilibration and subsequent non-equilibrium computation. We notice that the above MD results are in a good agreement with the continuous model for single fins if *mL*/2 = 0.28 (see also Figure 8). Hence, this enables us to estimate the thermal boundary conductance *α*_st _between SWNT and water with the help of Equation (15):(19)

The thermal conductivity *λ *has been independently computed by means of the technique illustrated in the sections above for the SWNT alone in a vacuum. Results for a nanofin with *L *= 14 nm are reported in Table 2. We stress that heat flux computed by time averaging of the Nosé-Hoover parameter *ξ *(see Equation (12)) is also in excellent agreement with the value predicted by the continuous model through Equation (18). For instance, with the above choice *mL*/2 = 0.28, for (5, 5) SWNT with *L *= 10 nm, *L*_NH _= 2 nm in a box 5 × 5 × 14 nm^3 ^we have: - 〈*ξ*〉 *N*_f _*k*_b _*T *= 3.11 × 10^-8 ^W while(20)

We stress that *L*_NH _is the axial length of the outermost carbon atom rings coupled to a thermostat at each end of a nanotube. Finally, a useful parameter when studying fins is the *thermal efficiency *Ω, expressing the ratio between the exchanged heat flux *q *and the ideal heat flux *q*_id _corresponding to an isothermal fin with *T *(*x*) = *T*(0), ∀*x *∈ [0, *L*] [44]. In our case, we find highly efficient nanofins:(21)

### Transient simulations

**Figure 9:**
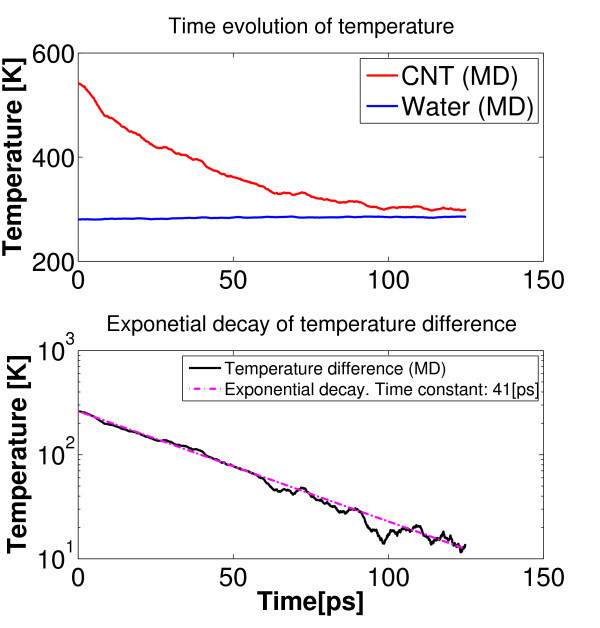
**Color online. Transient simulations: temperature evolution as predicted by NVE molecular dynamics**. Best fitting of exponential decay of the temperature difference *T*_CNT _- *T*_w _is achieved by choosing *τ*_d _= 41 ps.

The value of thermal boundary conductance between water and a SW-CNT has been assessed by transient simulations as well. Results by the latter methodology are denoted as *α*_tr _to distinguish them from the same quantities (*α*_st _) in the above section. In this study, the nanotube was initially heated to a predetermined temperature *T*_hot _while water was kept at *T*_w _*< T*_hot _(using in both cases Nosé-Hoover thermostatting for 0.6 ns). Next, an NVE MD (ensemble where number of particle N, system volume V and energy E are conserved) were performed, where the entire system (SWNT plus water) was allowed to relax without any temperature and pressure coupling. Under the assumption of a uniform temperature field *T*_CNT _(*t*) within the nanotube at any time instant *t *(i.e., Biot number Bi < 0.1), the above phenomenon can be modeled by an exponential decay of the temperature difference (*T*_CNT _- *T*_w _) in time, where the time constant *τ*_d _depends on the nanotube heat capacity *c*_T _and the thermal heat conductance *α*_tr _at the nanotube-water interface as follows (see Figure 9):(22)

In our computations, based on [20], we considered the heat capacity per unit area of an atomic layer of graphite *c*_T _= 5.6 × 10^-4 ^(J·m^-2^·K^-1^).

The values of *τ*_d _and *α*_tr _have been evaluated in different setups, and results are reported in the Table 2. Numerical computations do predict pretty high thermal conductance at the interface (order of 10^7 ^W·m^-2^·K^-1^) with a slight tendency to increase with both the tube length and diameter. It is worth stressing that values for thermal boundary conductance obtained in this study are consistent with both experimental and numerical results found by others for SW-CNTs within liquids [20,45]. However, since the order of magnitude of these results is extremely higher than that involved in macroscopic applications, it may appear as an artifact. Actually, it is quite simple to realize that continuum-based models diverge in case of nanometer dimensions, because of the effects of singularity. Hence, continuum-based predictions may lead to even higher thermal conductances, and they are not even upper bounded, which is clearly unphysical. For example, let us consider the ideal case of a circular cylinder (with diameter *D *and length *L*) centered in a square solid of equal length, as reported in Table 3.12 of [19]. The value of thermal boundary conductance can be put into relation with the heat conduction shape factor (CSF) *S*_f _as follows:(23)

where(24)

and *λ*_w _is the thermal conductivity of the medium, while the square box has dimensions *w *× *w *× *L*. Let us consider the following example, corresponding to the row '(5, 5), BAD-LJ (sol)' in Table 2. Assuming *λ*_w _= 0.58 (W·m^-1^·K^-1^), *D *= 0.68 nm, *w *= 4 nm, it yields *α*_csf _= 9.2 × 10^8 ^W m^-2 ^K^-1^. The analytic results are even larger than those obtained by the steady-state simulation (usually larger than those obtained by the transient method). Moreover, the continuum-based formula prescribes that thermal conductance (weakly) diverges by reducing the cylinder diameter. On the contrary, MD simulations is in line with the expectation of a bounded thermal boundary conductance. In fact, in agreement with others [45], we even observe a slight decrease with the tube diameter.

We point out that neither the steady-state method nor the transient method fully reproduce the setup described by the analytic formula (23). In fact, in the steady-state method, the entire water bath is thermostatted (while in the analytic formula, only the water boundaries are thermostatted) and, in the transient method, the water temperature changes in time (while the analytic formula is derived under steady-state condition). Nevertheless, from the technological point of view, the above results are in line with the basic idea that high aspect-ratio nanostructures (such as CNTs) are suitable candidates for implementing the above idea of *nanofin*, and thus can be utilized for exploiting advantageous heat boundary conductances.

## Conclusions

In this study, we first investigated the thermal conductivity of SW-CNTs by means of classical non-equilibrium MD using both simplified one-dimensional and fully three-dimensional models. Next, based on the latter results, we have focused on the boundary conductance and thermal efficiency of SW-CNTs used as nanofins within water. More specifically, toward the end of computing the boundary conductance *α*, two different approaches have been implemented. First, *α *= *α*_st _was estimated through a fitting procedure of results by steady-state MD simulations and a simple one-dimensional continuous model. Second, cooling of SWNT (at *T*_CNT _) within water (at *T*_w_) was accomplished by NVE simulations. In the latter case, the time constant *τ*_d _of the temperature difference (*T*_CNT _- *T*_w_) dynamics enables us to compute *α *= *α*_tr_. Numerical computations do predict pretty high thermal conductance at the interface (order of 10^7 ^W·m^-2^·K^-1^), which indeed makes CNTs ideal candidates for constructing nanofins. We should stress that, consistently with our results *α*_st _*> α*_tr_, it is reasonable to expect that *α*_st _represents the upper limit for the thermal boundary conductance, because (in steady-state simulations) water is forced by the thermostat to the lowest temperature at any time and any position in the computational box. Finally, it is worthwhile stressing that, following the suggestion in [46], all the results of this study can be generalized to different fluids using standard non-dimensionalization techniques, upon a substitution of the parameterization (ϵ_CO_, *σ*_CO_) representing a different Lennard-Jones interaction between SWNT and fluid molecules.

## Methods

The CNTs geometries simulated in this article were generated using the program Tubegen [39], while water molecules were introduced using the *SPC/E *model implemented by the *genbox *package available in GROMACS [38]. Numerical results in this study are based on non-equilibrium MD where the all-atom forcefields OPLS-AA is adopted for modeling atom interactions. Visualization of simulation trajectories is accomplished using VEGA ZZ [47].

## Abbreviations

CNTs: carbon nanotubes; GROMACS: GROningen MAchine for Chemical Simulations; MD: molecular dynamics; MMFF: molecular mechanics based on force fields; MW-CNTs: multi-walled carbon nanotubes; NEMD: non-equilibrium molecular dynamics; SW-CNTs: single wall CNTs; SWNT: single wall nanotube.

## Competing interests

The authors declare that they have no competing interests.

## Authors' contributions

Motivation for investigating the nanofin idea was initially provided by PA, and thereafter refined by an active interaction between both authors. All one-dimensional atomistic simulations and numerical experiments for assessing thermal boundary conductances *α *were performed by EC. Measurements of thermal boundary conductance through steady state (*α*_st_) and transient simulations (*α*_st_) were thought by PA, and EC, respectively. Computations of thermal conductivity with different combinations of interaction potentials, as reported in Figure 5, were performed by PA. Authors contributed equally in writing the present manuscript.
